# BGJ398, A Pan-FGFR Inhibitor, Overcomes Paclitaxel Resistance in Urothelial Carcinoma with FGFR1 Overexpression

**DOI:** 10.3390/ijms19103164

**Published:** 2018-10-15

**Authors:** Se Hyun Kim, Haram Ryu, Chan-Young Ock, Koung Jin Suh, Ji Yun Lee, Ji-Won Kim, Jeong-Ok Lee, Jin Won Kim, Yu Jung Kim, Keun-Wook Lee, Soo-Mee Bang, Jee Hyun Kim, Jong Seok Lee, Joong Bae Ahn, Kui-Jin Kim, Sun Young Rha

**Affiliations:** 1 Division of Hematology and Medical Oncology, Department of Internal Medicine, Seoul National University Bundang Hospital, Seoul National University College of Medicine, Seongnam 13620, Korea; sehyunkim@snubh.org (S.H.K.); ock.chanyoung@gmail.com (C.-Y.O.); skjmd0919@snubh.org (K.J.S.); maimatin83@snubh.org (J.Y.L.); jiwonkim@snubh.org (J.-W.K.); deafkeller@snubh.org (J.-O.L.); jwkim@snubh.org (J.W.K.); cong1005@snubh.org (Y.J.K.); hmodoctor@snubh.org (K.-W.L.); 65368@snubh.org (S.-M.B.); jhkimmd@snubh.org (J.H.K.); jslee@snubh.org (J.S.L.); 2 Department of Medicine, Graduate School of Yonsei University, Seoul 03722, Korea; 3 Medical Research Collaborating Center, Seoul National University Bundang Hospital, Seongnam 13605, Korea; r2218@snubh.org; 4 Division of Medical Oncology, Department of Internal Medicine, Yonsei University College of Medicine, Seoul 03722, Korea; vvswm513@yuhs.ac

**Keywords:** urothelial carcinoma, paclitaxel, FGFR, epithelial-to-mesenchymal transition, BGJ398, FGFR inhibitor, combination therapy

## Abstract

Paclitaxel (PTX) is commonly used to treat urothelial carcinoma (UC) after platinum-based chemotherapy has failed. However, single-agent taxane therapy is not sufficient to inhibit tumor progression and drug resistance in advanced UC. Epithelial-to-mesenchymal transition (EMT) induced by fibroblast growth factor receptor (*FGFR*)1 signaling has been proposed as a mechanism of PTX resistance, but it is unclear whether this can be overcome by *FGFR1* inhibition. The present study investigated whether *FGFR1* overexpression contributes to PTX resistance and whether *FGFR* inhibition can enhance PTX efficacy in UC. The effects of PTX combined with the *FGFR* inhibitor BGJ398 were evaluated in UC cell lines by flow cytometry; Western blot analysis; cell viability, migration, and colony forming assays; and RNA interference. PTX+BGJ398 induced cell cycle arrest and apoptosis in UC cells with mesenchymal characteristics was accompanied by downregulation of *cyclin D1* protein and upregulation of gamma-histone 2A family member X and cleaved poly(ADP-ribose) polymerase. Additionally, PTX+BGJ398 synergistically suppressed UC cell migration and colony formation via regulation of EMT-associated factors, while *FGFR1* knockdown enhanced the antitumor effect of PTX. These findings provide a basis for development of effective strategies for overcoming PTX resistance in UC through inhibition of *FGFR1* signaling.

## 1. Introduction

Urothelial carcinoma (UC) is the most common type of bladder cancer and a major cause of morbidity and mortality worldwide. Worldwide, it is estimated that over 400,000 new cases are diagnosed and 160,000 patients die from this disease annually [1]. More than half of bladder cancer patients are unfit for cytotoxic chemotherapy owing to poor performance status, decreased renal and cardiac function, and neuropathy [2]. Moreover, there are fewer clinical advances for UC than for other cancer types due to the difficulty in conducting clinical trials. Among patients with recurrent or metastatic disease, only 5% are alive 5 years after diagnosis.

Platinum-based combination chemotherapy has been the first-line treatment of locally advanced or metastatic bladder cancer over the past 30 years; typically, a combination of gemcitabine with cisplatin or carboplatin is used. Although immune checkpoint inhibitors are the current standard of care after failure of platinum-based chemotherapy, no standard cytotoxic chemotherapy regimens have been established [3]. There is therefore an urgent need for therapeutic approaches that inhibit the progression of bladder cancer while alleviating drug resistance to improve disease prognosis.

Taxanes such as paclitaxel (PTX) and docetaxel are the most widely used cytotoxic agents after failure of platinum-based chemotherapy for UC [4]. About 10% of patients respond to PTX monotherapy but this is generally short-lived, with a median progression-free survival of 2–3 months. Thus, single-agent taxane therapy is not sufficient to block tumor progression and overcome primary drug resistance in advanced UC. Clarifying the mechanisms of drug resistance in UC will be useful for establishing more effective drug combinations.

The activation of the fibroblast growth factor receptor (*FGFR*) signaling pathway plays an important role in UC development and progression [5]. For example, over half of non-muscle invasive UCs harbor activating point mutations in *FGFR3*, and 5% of cell lines and tumors have chromosomal translocations that generate *FGFR3* fusion proteins. A preclinical study demonstrated a link between *FGFR1* overexpression and increased cell proliferation and invasion, although no mutations were reported [6]. *FGF2* stimulation of *FGFR1β* in cultured normal human urothelial cells activates mitogen-activated protein kinase (*MAPK*) signaling and phospholipase Cγ, which enhanced proliferation and inhibited apoptosis [6]. In addition, small molecule tyrosine kinase inhibitors and monoclonal antibodies targeting *FGFR* pathway components showed promising anti-tumor activity in UC both in vitro and in vivo [7,8,9].

Epithelial-to-mesenchymal transition (EMT) is an evolutionarily conserved reprogramming process that occurs during embryonic development and tissue repair [10]. EMT is characterized by downregulation of surface *E-cadherin* expression reflecting the loss of epithelial integrity and upregulation of mesenchymal markers such as vimentin. Many lines of evidence indicate that EMT of cancer cells increases metastasis and contributes to the emergence of drug resistance during anti-cancer treatment. EMT in UC cells is triggered by *FGF2* via *FGFR1* signaling [8,11]. UC cell lines overexpressing *FGFR1* and *FGF2* also show strong expression of mesenchymal markers such as zinc finger E-box binding homeobox (*ZEB*)1 and *vimentin* [8].

EMT induced by *FGFR1* signaling is considered as the principal mechanism of metastasis and drug resistance in breast, lung, and prostate cancers [12,13,14,15,16]. However, it is not known whether inhibiting *FGFR1* can overcome PTX resistance in bladder cancer cell lines overexpressing *FGFR1*. To address this issue, this study examined whether *FGFR1* overexpression contributes to PTX resistance and whether *FGFR* inhibition enhances PTX efficacy in UC.

## 2. Results

### 2.1. FGFR1 Overexpression Is Correlated with EMT and PTX Resistance in UC Cell Lines

**Figure 1:**
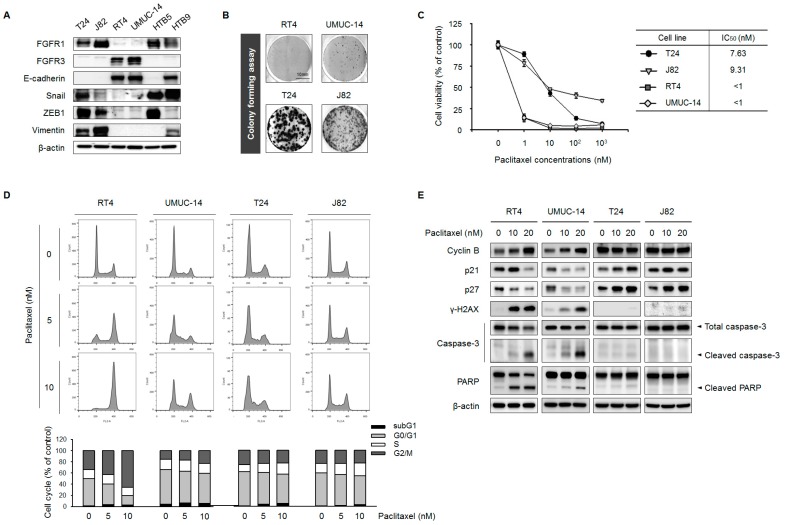
*FGFR1* expression is correlated with EMT features and PTX resistance in UC cell lines. (**A**) T24, J82, RT4, UMUC-14, HTB5, and HTB9 cells were evaluated basal expression of *FGFRs* and EMT-associated proteins by Western blotting; *β-actin* served as a loading control. (**B**) Colony formation assay. T24, J82, RT4, and UMUC-14 cells were grown for 7 days, then stained with Coomassie Brilliant Blue and counted. (**C**) T24, J82, RT4, and UMUC-14 cells were treated with 0, 1, 10, 100, and 1000 nM PTX for 3 days. IC_50_ values were calculated using CalcuSyn (BioSoft, Ferguson, MO, USA). Data represent the mean ± standard deviation of five replicates. (**D**) Cell cycle analysis by propidium iodide staining and flow cytometry. A total of 1 × 10^6^ cells were seeded in 60-mm plates and treated with 0, 5, and 10 nM PTX for 48 h. Data are presented as histograms (blue, G0/G1 phase; green, S phase, and red, G2/M phase). (**E**) *Cyclin B*, *p21*, *p27*, *γ-H2AX*, *caspase-3*, and *PARP* expression in T24, J82, UMUC-14, and RT4 cells, as determined by Western blotting; *β-actin* served as the loading control.

To investigate the correlation between *FGFR* expression and EMT features, we evaluated the expression of *FGFR1*, *FGFR3*, *E-cadherin*, *Snail*, *ZEB1*, and *vimentin* in six UC cell lines by Western blotting. In each of the cell lines, *FGFR1* and *FGFR3* were expressed in non-overlapping patterns; moreover, T24 and J82 cell lines expressing high levels of *FGFR1* showed prominent expression of the mesenchymal markers *Snail*, *ZEB1*, and *vimentin* (Figure 1A). In contrast, RT4 and UMUC-14 cells had high levels of *FGFR3* and *E-cadherin* but weak *Snail*, *ZEB1*, *vimentin*, and *FGFR1* expression. HTB5 and HTB9 cells did not exhibit distinct characteristics. Thus, T24 and J82 are mesenchymal-type whereas RT4 and UMUC-14 are epithelial-type cell lines, as previously reported [8]. We selected T24, J82, RT4, and UMUC-14 cell lines for further analysis.

Given that EMT is associated with tumor progression and drug resistance [17,18], we speculated that T24 and J82 cells would be more tumorigenic and drug-resistant than RT4 and UMUC-14 cells. We tested this hypothesis with the colony formation assay and cell viability assay. In colony formation assay, T24 and J82 cells showed more aggressive growth than RT4 and UMUC-14 cells (Figure 1B). To examine the effect of PTX on UC cell viability, T24, J82, RT4, and UMUC-14 cells were treated with different concentrations of PTX for 24 h. The half-maximal inhibitory concentrations (IC_50_) were higher for T24 (7.63 nM) and J82 (9.31 nM) cells than for RT4 (<1 nM) and UMUC-14 (<1 nM) cells (Figure 1C), suggesting that mesenchymal-type UC cells are more resistant to PTX than the epithelial-type cells.

Several studies have demonstrated that PTX induces cell cycle arrest via regulation of mitosis, leading to apoptosis [19,20,21]. To determine whether the cell cycle was altered by PTX treatment, we carried out flow cytometry analysis of UC cell lines. PTX treatment for 24 h increased the percentage of RT4 and UMUC-14 cells in G2/M phase and decreased that of cells in G0/G1 phase (Figure 1D). On the other hand, the G2/M phase fraction was reduced whereas the S phase fraction was increased in T24 and J82 cells treated with PTX.

We next investigated the mechanism of PTX-induced cell cycle changes in T24, J82, RT4, and UMUC-14 cell lines. Cell cycle inhibition by PTX was accompanied by upregulation of cyclin B1 and downregulation of *p21* and *p27* protein expression in RT4 and UMUC-14 cells (Figure 1E). Interestingly, expression of gamma-histone 2A family member X (*γ-H2AX*), *cleaved caspase-3*, and cleaved poly(ADP-ribose) polymerase (*PARP*)—which are DNA damage-induced pro-apoptosis markers [22,23]—was markedly increased 24 h after PTX treatment in RT4 and UMUC-14 cells. However, consistent with the results of the cell cycle analysis, PTX did not alter the expression of the G2/M phase cell cycle regulatory factor *cyclin B1* in T24 and J82 cells. We also found that PTX had opposite effects on *p21* and *p27* expression in T24 and J82 cells as compared to RT4 and UMUC-14 cells. Moreover, treatment with 20 μM PTX did not affect the expression of DNA damage-associated markers in T24 and J82 cells. These data provide additional evidence that mesenchymal-type T24 and J82 cell lines, which are characterized by *FGFR1* overexpression, exhibit increased resistance to PTX.

### 2.2. Expression Levels of FGFR1 and EMT Markers Are Correlated

**Figure 2:**
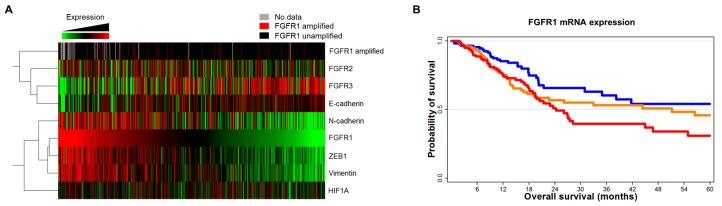
*FGFR1* expression is correlated with EMT marker levels in TCGA data. (**A**) Gene expression and alteration profiles from TCGA cohort (http://www.cbioportal.org). (**B**) Survival analysis according to *FGFR1* mRNA expression (red: upper third, orange: mid third, Blue: lower third).

To investigate the correlation between *FGFR1* and EMT marker expression in UC, we analyzed mRNA expression and survival data from The Cancer Genome Atlas (TCGA) using cBioPortal (http://cbioportal.org) [24]. The heat map showed a good correlation between high mRNA levels of *FGFR1* and of mesenchymal markers such as *N-cadherin* (encoding cadherin 2), *ZEB1*, and *vimentin* (Figure 2A)*.* In the univariate survival analysis, higher *FGFR1* mRNA expression was associated with reduced overall survival in UC patients (Figure 2B). The estimated hazard ratios for overall survival of patients with lower-third expression (*n* = 118) vs. middle-third expression (*n* = 125) and lower-third expression vs. upper-third expression (*n* = 125) were 1.47 (95% confidence interval [CI]: 0.94–2.28, *p* < 0.001) and 1.74 (95% CI: 1.13–2.70, *p* < 0.001), respectively.

### 2.3. FGFR Inhibition Overcomes PTX Resistance in UC Cell Lines

**Figure 3:**
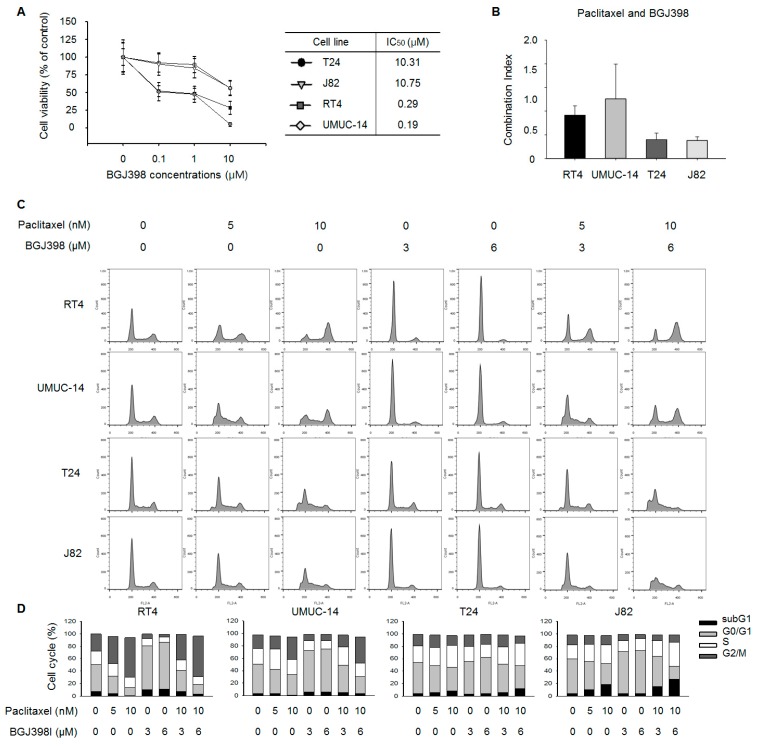
*FGFR* inhibition enhances PTX-induced cell cycle arrest in mesenchymal-type UC cell lines. (**A**) T24, J82, RT4, and UMUC-14 cells were treated with 0, 0.1, 1, and 10 μM BJG398 for 3 days. IC_50_ values were calculated using CalcuSyn (BioSoft, Ferguson, MO, USA). Data represent mean ± standard deviation of five replicates. (**B**) Cells were exposed to increasing concentrations of PTX and BGJ398 combinations at a fixed ratio. Viable cells were assayed and CI was calculated using CalcuSyn software. Lines above each bar represent CI for that dose combination (*n* = 3). (**C**) Cell cycle distribution and apoptosis were analyzed by propidium iodide staining and flow cytometry. A total of 1 × 10^6^ cells were seeded in a 60-mm plate and treated with indicated concentrations of PTX and BGJ398 for 48 h (*n* = 3). (**D**) Quantification of cell cycle distribution of T24, J82, RT4, and UMUC-14 cells.

BGJ398 is an orally available selective inhibitor of *FGFR1, FGFR2*, and *FGFR3* that has been shown to potently inhibit tumor cell proliferation and tumor growth in various cancer models harboring genetic alterations in *FGFR1/2/3*. We first carried out a cell viability assay to determine the IC_50_ of BGJ398 and found that the values for mesenchymal-type T24 (10.31 nM) and J82 (10.75 nM) cells were higher than those for epithelial-type RT4 (0.29 nM) and UMUC-14 (0.19 nM) cells (Figure 3A).

We next investigated the synergistic potential of PTX combined with BGJ398 according to the combination index (CI) theorem of Chou-Talalay [25] using CalcuSyn software [26]; by definition, CI values <1, =1, and >1 indicate synergy, additivity, and antagonism, respectively. Although mesenchymal-type T24 and J82 cells showed relative resistance to BGJ398 compared to epithelial-type UC cell lines, those cell lines showed a synergistic effect to the combination treatment of PTX and BGJ398 (CI value < 1; Figure 3B and Figure S1).

We performed a cell cycle analysis to determine whether PTX acts synergistically with BGJ398 to alter the cell cycle. Treatment with PTX alone increased the G2/M fraction of RT4 and UMUC-14 cells, while BGJ398 induced cell cycle arrest at G0/G1 (Figure 3C,D). Interestingly, no such synergistic effect on subG1 phase was observed in these cells upon treatment with both PTX and BGJ398. These results demonstrate that the combination drug treatment does not have synergistic effects on epithelial-type UC cells.

Treatment with PTX alone caused S phase accumulation and decreased G0/G1 and G2/M fractions in T24 and J82 cell lines, while BGJ398 application increased the proportion of cells in G0/G1 phase. Combined treatment with PTX and BGJ398 markedly increased the subG1 population, an effect that was more pronounced in J82 cells than in T24 cells.

**Figure 4:**
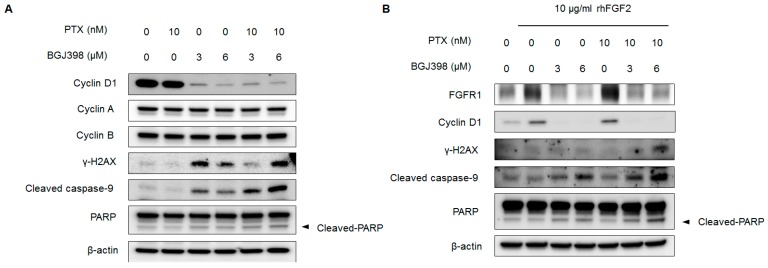
PTX and BGJ398 synergistically inhibit *cyclin D1* expression and stimulate apoptosis-associated proteins in J82 cell line. (**A**) Cells were treated with indicated concentration of PTX and BGJ398 for 24 h. Expression of *cyclin D1*, *cyclin A*, *cyclin B*, *γ-H2AX*, *caspase-9*, *PARP*, and *β-actin* (loading control) was analyzed by Western blotting. (**B**) *FGFR* signaling was activated with 10 μg/mL rhFGF2 for 3 h prior to treatment with indicated concentrations of PTX and BGJ398.

To investigate mechanistic basis for the synergistic effect of PTX and BGJ398, we examined whether treatment with both PTX and BGJ398 altered the expression of cell cycle regulators and pro-apoptosis factors in J82 cells by Western blotting. Application of 10 nM PTX along with 6 μM BGJ398 reduced *cyclin D1* but not *cyclin A* and *cyclin B* levels (Figure 4A). On the other hand, pro-apoptosis markers including *γ-H2AX*, *cleaved caspase-9*, and *cleaved PARP* were upregulated by this drug combination.

Recent data have suggested that *FGFR* signaling activates signaling pathways that promote cell cycle progression and inhibit apoptosis [27]. We therefore examined whether treatment with PTX, BGJ398, or their combination alters cell cycle- and apoptosis-related proteins upon activation of *FGFR* signaling in mesenchymal-type UC cells. Stimulation with recombinant human (rh)FGF2 only modestly increased *FGFR1* and cyclin D1 expression in J82 cells relative to the control (Figure 4B), which is consistent with a previous report [6]. Application of BGJ398 alone or in combination with PTX derepressed *FGFR1* and cyclin D1, whereas PTX by itself did not alter *FGFR1* and cyclin D1 expression. Interestingly, although rhFGF2 activated *FGFR* signaling in J82 cells, the combination of PTX and BGJ398 synergistically induced the expression of pro-apoptosis markers including *γ-H2AX*, *cleaved caspase-9*, and *cleaved PARP*, which is consistent with the data shown in Figure 4A. These results suggest that in UC cells exhibiting mesenchymal features, PTX by itself cannot stimulate DNA damage-induced apoptosis due to emergence of the EMT phenotype. However, in conjunction with *FGFR* inhibitor, PTX synergistically promotes apoptosis in these cells.

### 2.4. PTX Combined with BGJ398 Reduces the Migratory Capacity of Mesenchymal-Type UC Cells

**Figure 5:**
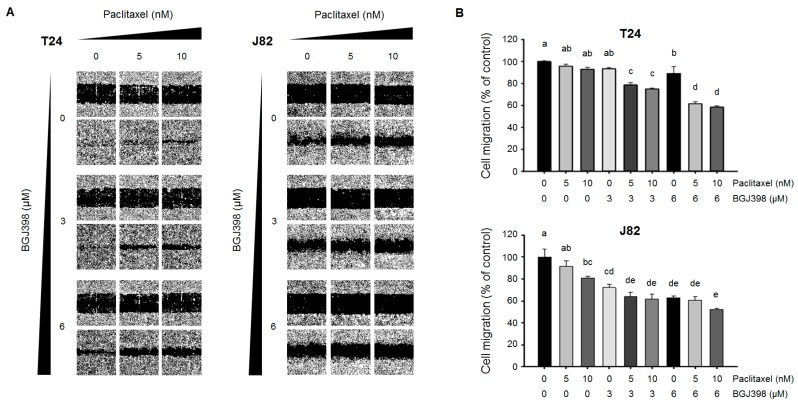
Evaluation of migratory potential of PTX and/or BGJ398 in mesenchymal-type UC cell lines. Cells were treated with 10 ng/mL rhEGF and 10 ng/mL rhFGF2. (**A**) Cell migration was assessed with the wound healing assay. Representative images of the scratched areas at different time points are shown. PTX+BGJ398 reduced cell migration rate in T24 and J82 cell lines. (**B**) Wound closure at 16 or 24 h after treatment as a percentage of control cell migration in T24 and J82 cells, respectively. Migrated cells were quantified with ImageJ software. Different letters (a, b, c, d) indicate significant differences (*p* < 0.05).

The above results indicate that combined treatment with PTX and BGJ398 exerts potent anti-tumor activity and overcomes PTX resistance in mesenchymal-type UC cells. An early step of tumor metastasis is an increased migratory capacity of cancer cells. We therefore investigated whether PTX combined with BGJ398 can inhibit UC cell migration. Epidermal growth factor receptor (*EGFR*) and downstream signaling are thought to be involved in EMT [11,28,29,30]. Moreover, rhEGF and rhFGF2 are known to enhance cell proliferation and migration. Therefore, the migration of mesenchymal-type UC cells was stimulated by application of rhEGF and rhFGF2. The T24 and J82 cell lines were also treated with mitomycin C to eliminate the effect of proliferation on cell migration. PTX and BGJ398 each inhibited the migration of T24 and J82 cells (Figure 5A,B). However, the combined treatment significantly reduced the rate of migration compared to each drug alone or PTX combined with a low concentration of BGJ398.

**Figure 6:**
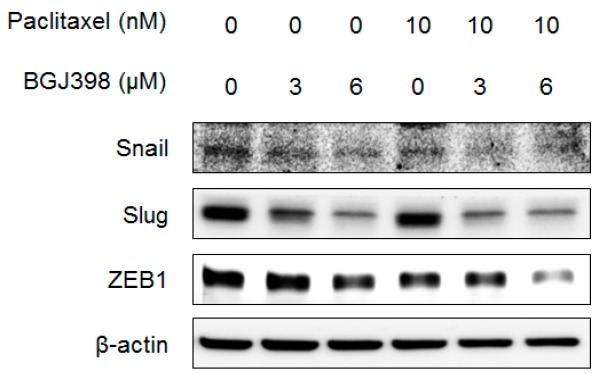
PTX and BGJ398 combination treatment synergistically decreases the protein levels of EMT inducers *Snail*, *Slug*, and *ZEB1*.

We next investigated whether PTX and BGJ398 affects cell migration via regulation of EMT-associated molecules by western blot analysis. The expression of the EMT induces *Snail*, *Slug*, and *ZEB1* was slightly reduced in T24 cells treated with PTX or BGJ398 alone (Figure 6); a greater decrease was observed upon application of PTX combined with BGJ398. These results support that the combination of PTX and BGJ398 inhibit UC cell migration by suppressing inducers of EMT.

### 2.5. BGJ398 Enhances the Effect of PTX on Colony Formation by Mesenchymal-Type UC Cells

**Figure 7:**
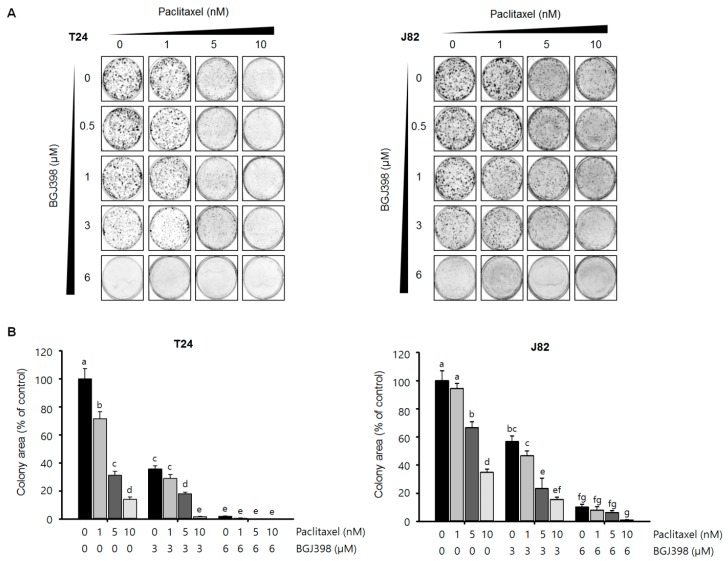
BGJ398 synergistically enhances the inhibitory effect of PTX on the colony forming ability of mesenchymal-type UC cell lines. (**A**) Colony size and number were reduced in a concentration-dependent manner upon combined treatment with BGJ398 and PTX. (**B**) Quantification of colonies. Different letters (a, b, c, d) indicate significant difference (*p* < 0.05).

The second step of metastasis after migration from the primary lesion is the formation of colonies at distal sites. Although combined treatment with PTX and BGJ398 markedly reduced the migratory capacity of mesenchymal-type UC cells, inhibiting colony formation by these cells is equally critical for preventing metastatic progression. To determine whether PTX+BGJ398 exerts this effect, colonies formation by T24 and J82 cells was examined by light microscopy. Treatment with the highest concentration of PTX (10 nM) combined with BGJ398 (6 μM) notably reduced colony number and size as compared to PTX or BGJ398 alone or application of low concentrations of PTX (<5 nM) combined with BGJ398 (<3 μM) (Figure 7A). Quantification of the colony forming area revealed that simultaneous application of the highest concentrations of PTX and BGJ398 prevented the formation of T24 and J82 cell colonies as compared to the other treatment groups (Figure 7B). Thus, combined treatment with PTX and BGJ398 not only induces tumor cell apoptosis but may also block metastatic progression by suppressing *FGFR1*-mediated migration and colony formation of mesenchymal-type UC cells.

### 2.6. Blocking FGFR1 Signaling Stimulates Apoptosis in PTX-Resistant UC Cells

**Figure 8:**
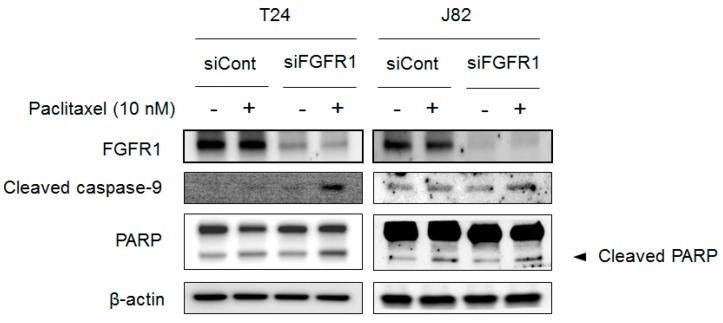
*FGFR1* knockdown sensitizes mesenchymal-type UC cells to PTX-induced DNA damage. Expression levels of pro-apoptotic proteins including *caspase-9* and *cleaved PARP* in T24 and J82 cell lines were evaluated by Western blotting.

Although BGJ398 enhanced the anti-tumor effect of PTX, it is unclear whether the synergistic effect of PTX and BGJ398 is mainly derived from the inhibition of *FGFR1* or its other target molecules. We therefore blocked *FGFR1* expression in T24 and J82 cells by RNA interference (RNAi) to assess the dependence of PTX sensitivity on apoptosis-associated markers. Compared to vehicle-treated control cells, *FGFR1* expression was downregulated by *FGFR1* knockdown in both cell lines as shown (Figure 8); this was accompanied by upregulation of apoptosis-associated markers including *cleaved caspase-9* and *cleaved PARP*. Thus, PTX resistance in UC cells exhibiting mesenchymal characteristics can be overcome by blocking *FGFR1* expression.

## 3. Discussion

Aberrant *FGFR* expression is associated with drug resistance [14], increased tumor cell proliferation, and survival through activation of *MAPK* and cyclin D1 [6]. Given the important functions of *FGF* signaling in various malignancies, therapeutic strategies that target *FGFR* could enhance the efficacy of clinically available drugs [31,32,33,34]. Although PTX alone is insufficient to block the progression of advanced UC [35], taxanes show promising anti-tumor effects as components of multi-drug therapeutic regimens used to treat advanced UC patients [36,37]. However, there have been no preclinical studies that have investigated the efficacy of PTX combined with *FGFR* inhibitor for the treatment of UC.

In the present study, we investigated whether the pan-*FGFR* inhibitor BGJ398 can used suppress the growth of UC cells either alone or in combination with PTX, which remains the treatment of choice after platinum-based chemotherapy. Our results showed that UC can be grouped into two distinct subtypes according to *FGFR1* and *FGFR3* expression, previously reported [8,38]. We also found that UC cells from patient-derived tumors harboring *FGFR1* amplification highly expressed EMT markers including *ZEB1* and *vimentin*, possibly due to the persistence of drug effects. In most cases, the *FGFR1* overexpression is negatively correlated with increased *FGFR3* levels in UC. Consistent with clinical findings, we found that two (T24 and J82) of six UC cell lines expressed relatively high levels of *FGFR1*, *vimentin*, and *Slug*, whereas *E-cadherin* expression was absent as compared to *FGFR3*-positive UC cell lines. Interestingly, mesenchymal-type T24 and J82 cell lines were highly resistant to PTX or BGJ398 monotherapy compared to *FGFR3*-positive epithelial-type (i.e., RT4 and UMUC-14) UC cell lines. These results demonstrate that *FGFR1* expression is associated with mesenchymal features and resistance to PTX or BGJ398 monotherapy. We tested the ability of combined BGJ398 and PTX treatment to overcome PTX resistance in UC cell lines with *FGFR1* overexpression, and found that subG1 levels (apoptotic cell population) of BGJ398 markedly increased the sensitivity of the cells—including those exhibiting mesenchymal characteristics—to PTX.

*FGFR1* is a receptor tyrosine kinase that regulates cell growth, survival, migration, and apoptosis [39,40]. Although PTX alone was insufficient to induce apoptosis caused by mitotic inhibition, PTX combined with BGJ398 delayed cell cycle transition, which resulted in apoptosis in mesenchymal-type UC cell lines. A previous study showed that *cyclin D1* depletion caused DNA damage via modulation of *γ-H2AX* and *PARP* [41] and re-sensitized tumor cells to antitumor drug [42,43]. In the present study, the combination of PTX and BGJ398 more potently suppressed cyclin D1 expression compared to PTX or BGJ398 monotherapy, resulting in *γ-H2AX* and *PARP* activation. This is in partial agreement with a previous report in which application of the *FGFR* inhibitor PD173074 combined with PTX promoted cell cycle arrest and apoptosis in a different cancer model [44].

Aberrant *FGFR1* expression contributes to metastasis in prostate and lung cancers and hepatocellular carcinoma [45,46,47]. The first step of metastasis after EMT is migration of primary tumor cells into the microenvironment around the primary tumor. The migrated cells then undergo metastasis to distant organs. Several transcription factors including *Snail*, *Slug*, *ZEB1*, and *Twist* have been implicated in the regulation of EMT [48]. The results of our study showed that PTX combined with BGJ398 synergistically inhibited cell migration in the wound healing assay and colony formation via downregulation of *Snail*, *Slug*, and *ZEB1* in mesenchymal—type UC cell lines.

The RNAi experiments demonstrated that the *FGFR1* signaling plays a key role in the mechanism of PTX resistance in mesenchymal-type UC cells. UC with *FGFR1* activation and EMT features would have resistance to anti-cancer treatment, which can result in poor survival outcome compared to UC patients without *FGFR1* overexpression. In accordance with our TCGA data analysis, Lim et al. suggested that about 45% of UC patients with *FGFR1* overexpression by immunohistochemistry had shorter OS with hazard ratio of 2.23 (95% confidence interval: 1.27–3.90, *p* = 0.006) [49].

Our results are in agreement with previous reports that cancers with *FGF* ligand and receptor overexpression exhibit a cancer stem cell-like phenotype, resistance to therapeutic agents, and enhanced metastasis [12,13,14,15,16]. Our results demonstrate that EMT features of tumor cells can be abolished by inhibiting *FGFR1* signaling by short interfering (si)RNA or small-molecule kinase inhibitors. BGJ398 strongly suppressed the dissemination of circulating tumor cells and metastasis in mice bearing orthotopically implanted mesenchymal-type UMUC-3 cells [8]. Non-invasive bladder cancer can become invasive and result in distant metastasis leading to death. Identifying biomarkers of this transition and preventing the acquisition of invasive and metastatic properties by tumor cells can potentially reduce the fatal progression of bladder cancer. Cyclooxygenase (*COX*)2 inhibitors are candidate agents for chemoprevention in non-invasive bladder cancer, since activation of *FGFR1* signaling has been shown to induce EMT in UC by promoting phospholipase C gamma-mediated upregulation of *COX2* [11].

In early clinical trials, BGJ398 showed good efficacy only in UC patients with *FGFR3* mutation or translocation [9]. Although the objective response rate of 25.4% was favorable, only 15–20% of UC patients harbor *FGFR3* alterations [9]. The important finding of this study is that *FGFR1* inhibition by BGJ398 restores PTX sensitivity in drug-resistant UC cell lines and decreases their metastatic potential. Given that the expression profiles of *FGFR1* and *FGFR3* are mutually exclusive, it is conceivable that BGJ398 and PTX combination treatment can be applied to not only in UC patients with *FGFR3* alteration, but also with *FGFR1* overexpression.

In conclusion, we investigated the mechanism of resistance to PTX in UC mediated by *FGFR1* and found that combined treatment with BGJ398 can enhance the efficacy of PTX in UC cell lines with mesenchymal features. Our results demonstrate that combination with *FGFR1*-targeted therapy improves the antitumor efficacy of standard cytotoxic chemotherapy, which is a strategy that warrants further investigation in clinical trials in selected UC patients with *FGFR1* overexpression.

## 4. Materials and Methods

### 4.1. Materials

PTX and BGJ398 were purchased from Selleckchem (Houston, TX, USA). The following antibodies against the following proteins were purchased from Santa Cruz Biotechnology (Dallas, TX, USA): cyclin D1 (sc-753), *cyclin B1* (sc-752), *cyclin A* (sc-751), *p27* (sc-1641), *PARP* (sc-8007), *β-actin* (sc-130656). Antibodies against *Snail* (cs#3879), *E-cadherin* (cs#3195), *vimentin* (cs#5741), *FGFR1* (cs#9740), *FGFR3* (cs#4574), *p21* (cs#2947), *caspase-3* (cs#9668), *γ-H2AX* (cs#2577), *β-actin* (cs#4970), and *cleaved caspase-9* (cs#7237) were purchased from Cell Signaling Technology (Danvers, MA, USA). rhFGF and rhEGF proteins were purchased from R&D Systems (Minneapolis, MN, USA). All chemicals and reagents used were of analytical and were obtained from commercial sources.

### 4.2. Cell Culture

UMUC-14, RT4, T24, J82, HTB5, and HTB9 cells were purchased from the Korean Cell Line Bank (Seoul, Korea). UMUC-14 cells were maintained in Minimum Essential Medium supplemented with 10% fetal bovine serum (FBS), 1% non-essential amino acid, and 1% penicillin/streptomycin (P/S) at 37 °C with 5% CO_2_. T24 cells were maintained in Roswell Park Memorial Institute 1640 medium with 10% FBS, and 1% P/S at 37 °C with 5% CO_2_. RT4, HTB5, and HTB9 cells were maintained in Dulbecco’s modified Eagle’s medium with 10% FBS and 1% P/S at 37 °C with 5% CO_2_.

### 4.3. Cell Viability Assay

Cell viability was assessed with the CellTiter-Glo Luminescent Cell Viability Assay (Promega, Madison, WI, USA) according to the manufacturer’s instructions. On day 0, 96-well plates were seeded with 5000 cells/well and incubated overnight. The next day (day 1), cells were treated with the appropriate compounds. On day 4, the plates were incubated for 60 min at room temperature and 100 μL of CellTiter-Glo reagent was added to each well, followed by mixing on an orbital shaker for 5 min. Luminescence was quantified on a standard plate luminometer (LMAX; Molecular Devices, Sunnyvale, CA, USA).

### 4.4. Analysis of Combination Index

Cells were seeded in 96-well plates at 3000 cells per well in a total volume of 100 μL media containing 10% FBS. The following day, cells were treated in pentaplicate with single agents and their fixed-ratio combination for 72 h over a 7-point, which was centered on the single-agent concentrations that inhibited viability by 50% (IC_50_). Cell viability was measured by the CellTiter-Glo Luminescent Cell Viability Assay (Promega, Madison, WI, USA) according to the manufacturer′s instructions. Combination index (CI) scores were calculated as previously described [25] using CalcuSyn software (Biosoft, Ferguson, MO, USA). This software uses the Chou-Talalay combination index method, which is based on the median-effect equation, itself a derivation from the mass-action law. For this analysis, BGJ398 was combined with PTX at a constant ratio determined by IC_50_ BGJ398/IC_50_ PTX. We entered the resulting proliferation data, along with the data obtained from single drug treatments, into CalcuSyn to determine a CI value for each combination point, which quantitatively defines synergy (CI < 1), additivity (CI = 1), and antagonism (CI > 1).

### 4.5. Western Blot Analysis

Cell lysates were clarified by centrifugation at 12,000× *g* for 20 min at 4 °C. The protein concentration in the supernatant was measured with the Bradford assay (BioLegend, San Diego, CA, USA); proteins (20–40 μg) were separated by sodium dodecyl sulfate polyacrylamide gel electrophoresis, and transferred to a polyvinylidene difluoride membrane (Bio-Rad, Hercules, CA, USA) that was blocked in blocking buffer containing 5% skim milk and then probed overnight with primary antibodies. Secondary antibodies conjugated with horseradish peroxidase (1:4000 dilution; Bio-Rad) were applied for 1 h. Immunoreactivity was detected by enhanced chemiluminescence (Biosesang, Seongnam, Korea) and a ChemiDoc Touch imager (Bio-Rad).

### 4.6. Cell Migration

Cells were seeded in 96-well plates and grown for 18 h. Confluent monolayers were gently scratched using a WoundMaker (Essen Bioscience, Ann Arbor, MI, USA). The cells were washed twice with phosphate-buffered saline (PBS) to remove floating cells and then incubated for various times (from 18 to 24 h) in growth medium supplemented with 10 ng/mL rhEGF, 10 ng/mL rhFGF2, and 10 μg/mL mitomycin C (to inhibit cell proliferation). Cell migration rate is expressed as the area of the scratch wound relative to total area of the cell-free region immediately after the scratch wound using IncuCyte Zoom (Essen Bioscience).

### 4.7. RNAi

T24 and J82 cells were transfected with control or *FGFR1* siRNA (Bioneer, Daejeon, Korea; ca. nos. SN-1003 and SC-1033, respectively) at a concentration of 100 nM using Lipofectamine 2000 reagent (Life Technologies, Carlsbad, CA, USA). After 24 h, the cells were treated with the appropriate compounds for 24 h and then harvested for analysis of protein expression.

### 4.8. Cell Cycle Analysis

Cells were seeded in 100-mm plates and grown overnight, then subjected to the appropriate treatment for 24 h. After trypsinization, the cells were washed twice in PBS, fixed overnight at 4 °C in ethanol, washed three times in PBS, and incubated in PBS containing 20 μg/mL propidium iodide and 100 μg/mL RNAse at 37 °C for 30 min. After washing in PBS, the cells were resuspended in 1 mL PBS and sorted on a FACSCalibur flow cytometer (BD Biosciences, Franklin Lakes, NJ, USA). Cell cycle distribution was determined using FlowJo software (Tree Star, Ashland, OR, USA).

### 4.9. Colony Formation Assay

Cells were seeded in 6-well plates and grown for 3 days before being subjected to the appropriate treatment for 4 days, with a medium change at regular time intervals. After 4 days of culture at 37 °C with 5% CO_2_, colonies were washed with PBS and stained with Coomassie Brilliant Blue for 30 min at room temperature, then washed with water and air-dried. The colonies were photographed using the ChemiDoc Touch (Bio-Rad) and counted using ImageJ software (National Institutes of Health, Bethesda, MD, USA).

### 4.10. Statistical Analysis

Statistical analyses were performed using SPSS v.12.0 software (SPSS Inc., Chicago, IL, USA). One-way analysis of variance was used for comparisons among groups. Significant differences between mean values were assessed with Duncan’s test. *p* < 0.05 was considered statistically significant.

## Supplementary Materials

Supplementary materials can be found at http://www.mdpi.com/1422-0067/19/10/3164/s1.
