# Supplementary figures and images for: BGJ398, A Pan-FGFR Inhibitor, Overcomes Paclitaxel Resistance in Urothelial Carcinoma with FGFR1 Overexpression
Source: Int J Mol Sci. 2018 Oct 15;19(10):3164. doi: 10.3390/ijms19103164 (PMC6214101; doi:10.3390/ijms19103164)

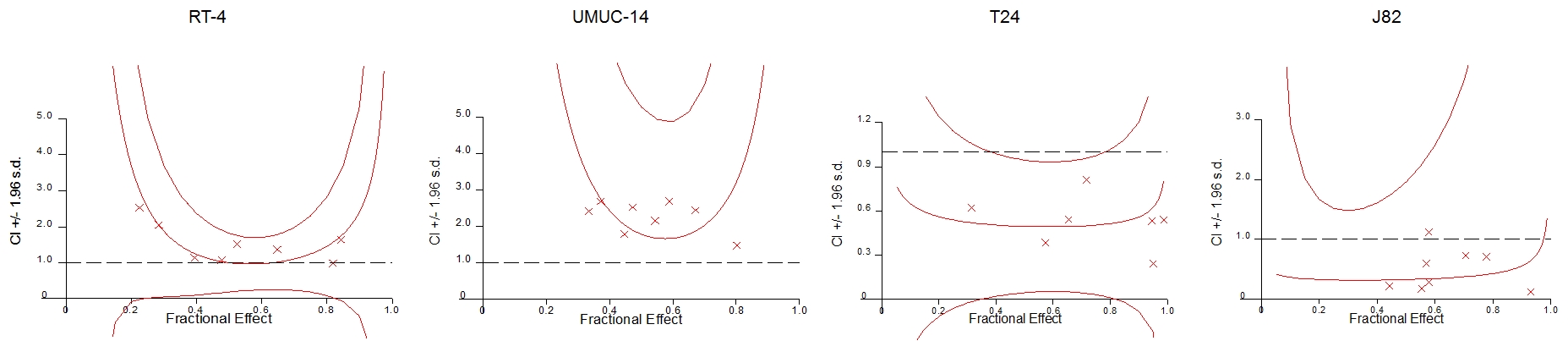

Supplement: Supplementary file 1 [file ijms-19-03164-s001.jpg]
